# Carrier Dynamics and Electro-Optical Characterization of High-Performance GaN/InGaN Core-Shell Nanowire Light-Emitting Diodes

**DOI:** 10.1038/s41598-017-18833-6

**Published:** 2018-01-11

**Authors:** Mohsen Nami, Isaac E. Stricklin, Kenneth M. DaVico, Saadat Mishkat-Ul-Masabih, Ashwin K. Rishinaramangalam, S. R. J. Brueck, Igal Brener, Daniel F. Feezell

**Affiliations:** 1 Center for High Technology Materials, University of New Mexico, Albuquerque, USA; 2 Center for Integrated Nanotechnologies, Sandia National Laboratories, Albuquerque, USA

## Abstract

In this work, we demonstrate high-performance electrically injected GaN/InGaN core-shell nanowire-based LEDs grown using selective-area epitaxy and characterize their electro-optical properties. To assess the quality of the quantum wells, we measure the internal quantum efficiency (IQE) using conventional low temperature/room temperature integrated photoluminescence. The quantum wells show a peak IQE of 62%, which is among the highest reported values for nanostructure-based LEDs. Time-resolved photoluminescence (TRPL) is also used to study the carrier dynamics and response times of the LEDs. TRPL measurements yield carrier lifetimes in the range of 1–2 ns at high excitation powers. To examine the electrical performance of the LEDs, current density–voltage (J-V) and light-current density-voltage (L-J-V) characteristics are measured. We also estimate the peak external quantum efficiency (EQE) to be 8.3% from a single side of the chip with no packaging. The LEDs have a turn-on voltage of 2.9 V and low series resistance. Based on FDTD simulations, the LEDs exhibit a relatively directional far-field emission pattern in the range of $\pm$15°. This work demonstrates that it is feasible for electrically injected nanowire-based LEDs to achieve the performance levels needed for a variety of optical device applications.

## Introduction

III-nitride based micro-light emitting diodes (µ-LEDs) are expected to become the next generation of pixel-level emitters in display technology. These displays include indoor/outdoor video walls, smart phones, tablets, televisions, smart watches, and head-mounted displays. µ-LEDs offer potential advantages compared to conventional organic LEDs (OLEDs) and liquid crystal displays (LCDs). Some advantages include higher brightness, higher transparency, longer lifetimes, lower power consumption, and shorter response times^1–4^. III-nitride based µ-LEDs exhibit a luminance of 10^5^ cd/m^2^, while the luminance of LCDs and OLEDs are 3000 cd/m^2^ and 1500 cd/m^2^, respectively^4^. Additionally, µ-LEDs exhibit nanosecond response times, in contrast to the millisecond and microsecond response times of LCDs and OLEDs, respectively^4^. The short response times of µ-LEDs pave the way for the next generation of indoor LED-based data communication, known as light-fidelity (Li-Fi)^5^. The brightness, etendue, and acceptable light efficiency of µ-LED displays are also strongly dependent on the far-field emission pattern of the individual devices^6^. A small far-field radiation angle of ±15–20° is critical in full-color LED displays^6,7^. The photonic crystal (PhC) effect has been used to engineer the far-field emission pattern of conventional planar LEDs, although processing of photonic crystal structures poses challenges^8–12^ Alternatively, µ-LEDs based on nanowire structures (nanowire-based LEDs) enable engineering of the far-field emission pattern using the periodic nature of the nanowires themselves. Indeed, a more directional far-field emission pattern has been achieved experimentally using nanowire-based LEDs^7,13^. In addition, nanowire-based emitters offer an approach to monolithically integrated RGB-based white LEDs and lasers^14–18^. These monolithically integrated multi-colored µ-LEDs can be achieved in a single growth by varying the pitch spacing between the nanowire pattern, potentially providing significant cost reduction due to fewer processing steps and consumed sources compared to pick-and-place approaches. In addition to display technology, RGB-based white µ-LEDs are preferred over phosphor-converted approaches for visible-light communication (VLC) systems with high data rates^19^ due to the slow response of the phosphors in conventional phosphor-converted white LEDs^20,21^. Dynamic color tuning is also achievable using a multi-color approach^22^, which could potentially lead to multiplexing within VLC over several wavelength channels. In addition to LEDs, III-nitride nanowires are promising candidates for the next generation of other optoelectronic and electronic devices such as high power transistors^23^, gas sensors^24,25^, DNA sensors^26,27^, visible-blind UV photodetectors^28,29^, solar cells^30,31^,and atomic force microscopy (AFM) probe tips^32^.

**Figure 1:**
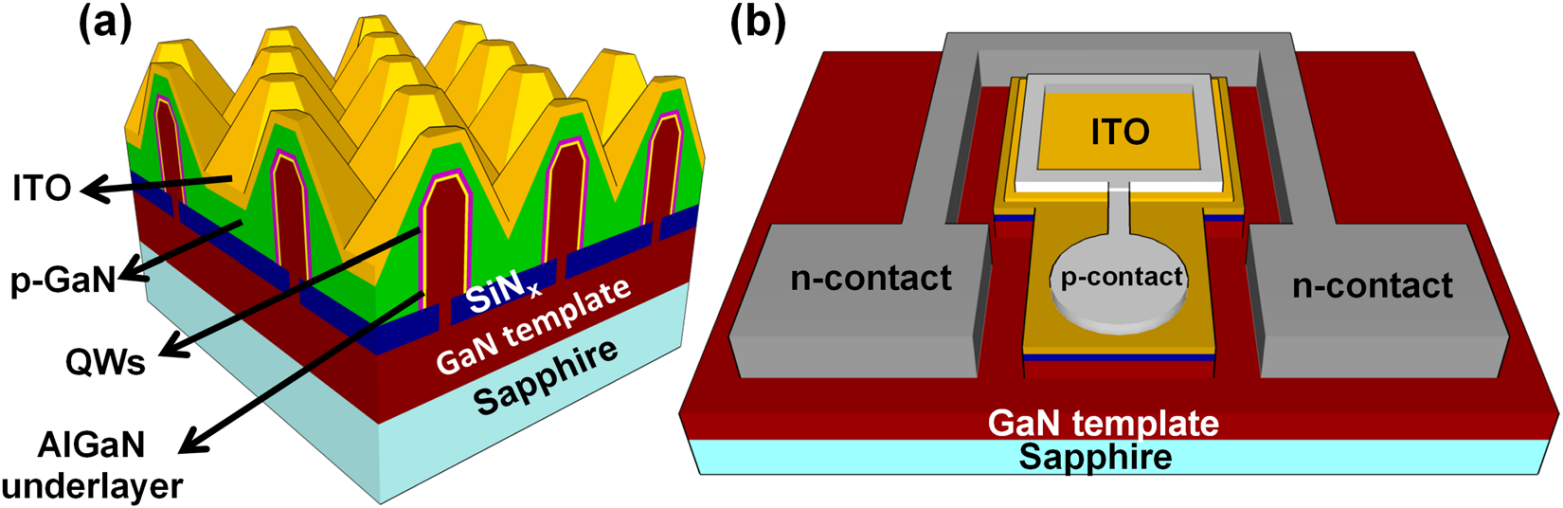
Schematic images of (**a**) device cross section and (**b**) device layout for the GaN/InGaN core-shell nanowire-based LEDs.

Figure 1 shows a schematic of a gallium nitride/indium gallium nitride (GaN/InGaN) core-shell nanowire-based LED. These LEDs offer several advantages over conventional planar structures, including lower dislocation density^33^ and substantially larger active region surface area (~4–8X) than their planar foot-print suggests^34^. Larger active region area leads to lower carrier density for a given drive current, which could mitigate the effects of LED efficiency droop. Lower efficiency droop for nanowire-based LEDs has been shown experimentally^35,36^. In addition, conventional c-plane GaN-based µ-LEDs suffer from internal polarization-related electric fields^37,38^ that hinder their radiative efficiency. Quantum wells (QWs) grown on nonpolar or semipolar planes have significantly reduced spontaneous and piezoelectric polarizations. Reduction of internal electric fields improves the radiative efficiency, reduces the carrier lifetime, and reduces the efficiency droop^39,40^. Despite promising results, the small area and high cost of free-standing GaN substrates are two main obstacles for the commercial adoption of nonpolar and semipolar LEDs. Alternately, nonpolar and semipolar devices based on nanostructures such as nano-micro walls^41–43^, nanowires^44,45^,and triangular-stripes^46–48^ grown on commercial c-plane sapphire substrates could potentially overcome these obstacles and enable RGB LEDs on a single chip.

The recombination lifetime is also a key parameter that determines the 3dB modulation speed of µ-LEDs. The 3dB modulation speed plays a critical role in the performance of µ-LEDs used for Li-Fi technology and VLC. The presence of internal electric fields reduces the spatial overlap of the electron-hole wave functions in polar c-plane QWs and increases the radiative recombination lifetime^49^. The recombination lifetimes of planar c-plane LEDs range from 4–20 ns at room temperature^50–52^. These recombination lifetimes correspond to 3dB modulation bandwidths of less than 50 MHz. However, a modulation speed of 1.1 GHz has been shown experimentally using GaN/InGaN core-shell nanowire-based LEDs^53^. Theoretical work has also predicted a range of tens of picoseconds for the carrier lifetime of plasmonic GaN/InGaN core shell nanowire-based LEDs. With proper engineering to increase light extraction, the plasmonic approach may enable LEDs that leverage the Purcell effect to operate at modulation speeds in the range of tens of GHz^54–57^.

The small dimensions of nanowires also help reduce the threading dislocation (TD) density in heteroepitaxial growth by virtue of dislocation bending toward the sidewalls of the nanowires^33^ and TD filtering using a dielectric mask^58^. Due to the small dimensions of nanowires, lateral strain relaxation on the nonpolar m-plane sidewalls allows higher levels of indium (In) incorporation during In_x_Ga_1−x_N/GaN (x = mole fraction of In) core-shell growth^59,60^. The higher growth rate of InGaN QWs grown on GaN nanowires, caused by a larger periodic spacing of the nanowires, also leads to higher levels of indium (In) incorporation during the core-shell growth^18^.

The three most common approaches for achieving GaN nanowires are catalyst-assisted, catalyst-free selective-area epitaxy (SAE) using *in-situ* deposition of a dielectric layer, and catalyst-free SAE using *ex-situ* deposition of a dielectric layer. The catalyst-assisted approach uses a vapor-liquid-solid (VLS) technique in either metalorganic chemical vapor deposition (MOCVD)^61,62^ or laser-assisted catalytic growth^63^. *In-situ* catalyst-free SAE may use either molecular beam epitaxy (MBE)^64^ or MOCVD^65^. *Ex-situ* catalyst-free SAE may also use either MBE^66–68^ or MOCVD^69–72^. The catalyst-assisted VLS growth technique using MOCVD does not enable well-controlled GaN nanowires and the metal catalyst is often incorporated as a deep level impurity^73^. Among catalyst-free techniques, the *ex-situ* approach is best suited to increase the homogeneity of the nanowires since the *in-situ* technique results in poor uniformity in the position and dimension of the nanowires. However, for the MBE *ex-situ* technique, the shadowing effect limits the growth of core-shell QWs^74–76^. Using the MOCVD *ex-situ* catalyst-free technique, the geometry of the nanowires is well controlled with a dielectric mask, the shadowing effect is absent, and no metal catalyst incorporates into the GaN. Therefore, the MOCVD *ex-situ* catalyst-free technique is a promising approach for growing controlled arrays of uniform, high-quality GaN nanowires.

Although there are numerous reports related to growth techniques for bottom-up core-shell nanowires using SAE, few of these reports demonstrate high-performance electrically-injected nanowire-based LEDs^36,44,45,77^. One of the most significant challenges in obtaining electrically injected devices is achieving efficient p-type doping in the core-shell nanowire LEDs. Previously, we developed a technique to improve the p-GaN growth in nanostructure-based LEDs^78^. This technique provides a path toward high-performance electrically-injected GaN/InGaN core-shell nanowire-based LEDs by improving the turn-on voltage and reducing the reverse-leakage current.

Here we present electrically-injected GaN/InGaN core-shell nanowire-based LEDs with among the highest performance levels reported thus far^36,44,45,77^. We perform a thorough investigation of the optical and electrical characteristics and compare the device performance to previously reported core-shell nanowire LEDs. In addition, we study the carrier dynamics of the LEDs to understand the response times, which are critical for predicting performance in VLC and Li-Fi systems. This work demonstrates that GaN/InGaN core-shell nanowire LEDs have the potential to reach the performance levels needed for a variety of lighting and display applications.

## Experimental growth and LED fabrication

**Figure 2:**
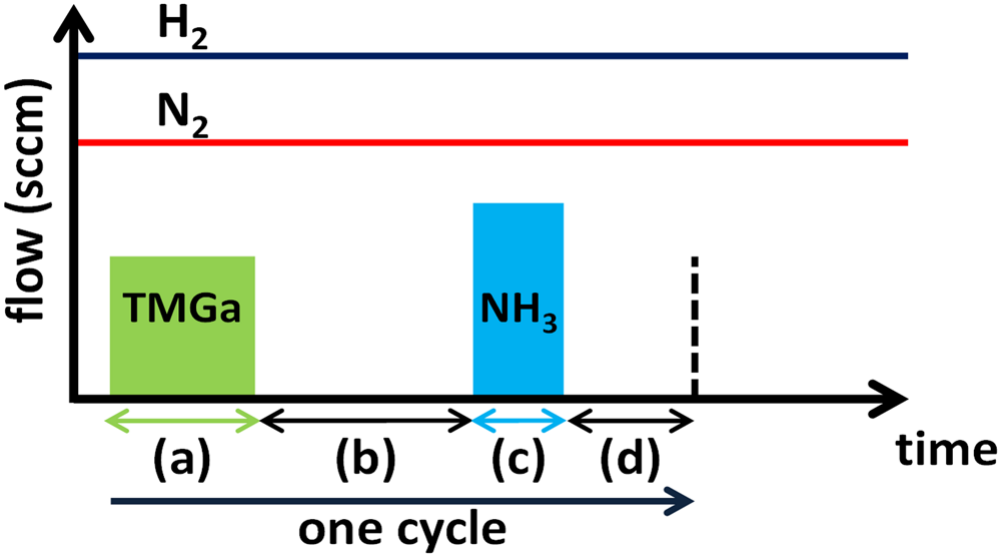
Schematic image of different steps in each cycle for pulsed-mode growth technique, (**a**) TMGa injection time, (**b**) TMGa interruption time, (**c**) NH_3_ injection time, (**d**) NH_3_ interruption time.

The GaN nanowires were grown on a selectively patterned c-plane GaN template on a sapphire substrate using a turbo disc VEECO p-75 MOCVD system. Initially, a 2-μm-thick GaN layer doped with silicon (n-GaN) was grown on a 2 inch single-side-polished sapphire substrate using conventional continuous-mode MOCVD and typical group III (TMGa) and group V (NH_3_) precursors. The wafer was then cleaned and 120 nm of SiN_x_ was deposited using plasma enhanced chemical vapor deposition (PECVD), followed by interferometric lithography^79^ and Cl_2_-based reactive-ion etching to pattern arrays of circular apertures of 400 nm diameter in the SiN_x_. The periodic spacing (pitch) between the apertures was 1 µm. A secondary contact lithography was performed to define the mesa size of the devices^34,46^. The samples were then cleaned in piranha etchant and loaded into the reactor for the nanowire growth. The GaN nanowires were grown using pulsed-mode MOCVD in an H_2_-N_2_ atmosphere. The chamber pressure was maintained at 90 Torr during the nanowire growth and the H_2_ and N_2_ flows were 3000 and 1000 sccm, respectively. 150 pulsed-mode cycles (see Fig. 2) were employed to grow the GaN nanowires. The growth temperature was held constant at 925 °C during the nanowire growth. The TMGa injection, TMGa interruption, NH_3_ injection, and NH_3_ interruption times were 18, 27, 6, and 15 seconds, respectively. The TMGa and NH_3_ flow rates were 26.7 µmol min^−1^ and 8 mmol min^−1^, respectively. The V/III ratio was 100. Under conventional continuous-mode MOCVD growth conditions with the relatively high V/III ratios used to grow GaN planar templates on sapphire substrates, SAE growth of GaN nanostructures predominately results in pyramidal structures limited by $\{10\bar{1}1\}$ semipolar planes^46^. As an alternative to the continuous-mode growth - where the nitrogen and gallium precursors are injected at the same time - Hersee *et al*.^69^ used a pulsed-mode technique for the controlled growth of GaN nanowires, wherein the group V (N) and group III (Ga) precursors are temporally pulsed (Fig. 2). The mechanisms of nanowire formation by the pulsed-mode growth technique were discussed later by Lin *et al*.^80^ and Jung *et al*.^70^. Recently, Choi *et al*.^81^ and Coulon *et al*.^72^ have also successfully demonstrated GaN nanowire growth using the continuous growth mode with a very low V/III ratio.

**Figure 3:**
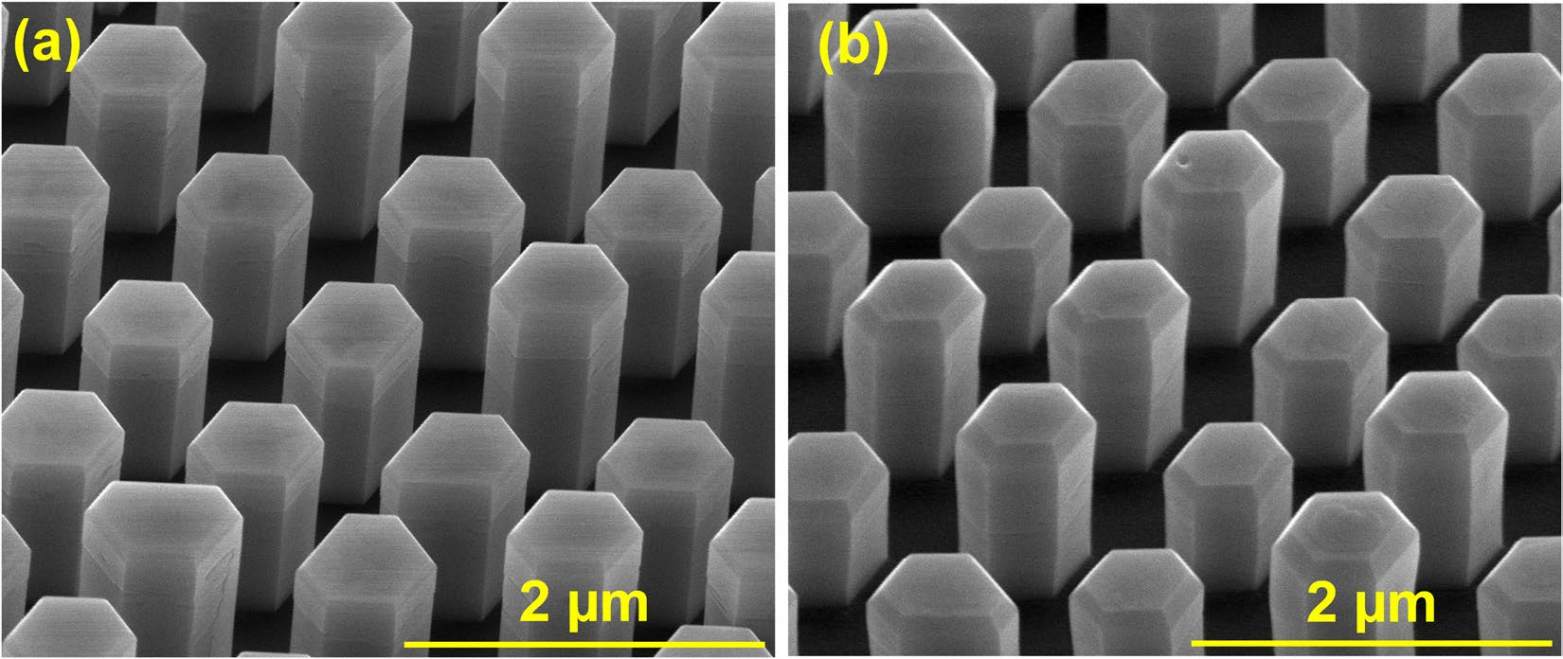
SEM images, at a 45-degree tilt, of GaN nanowires (**a**) before (**b**) after AlGaN underlayer growth.

**Figure 4:**
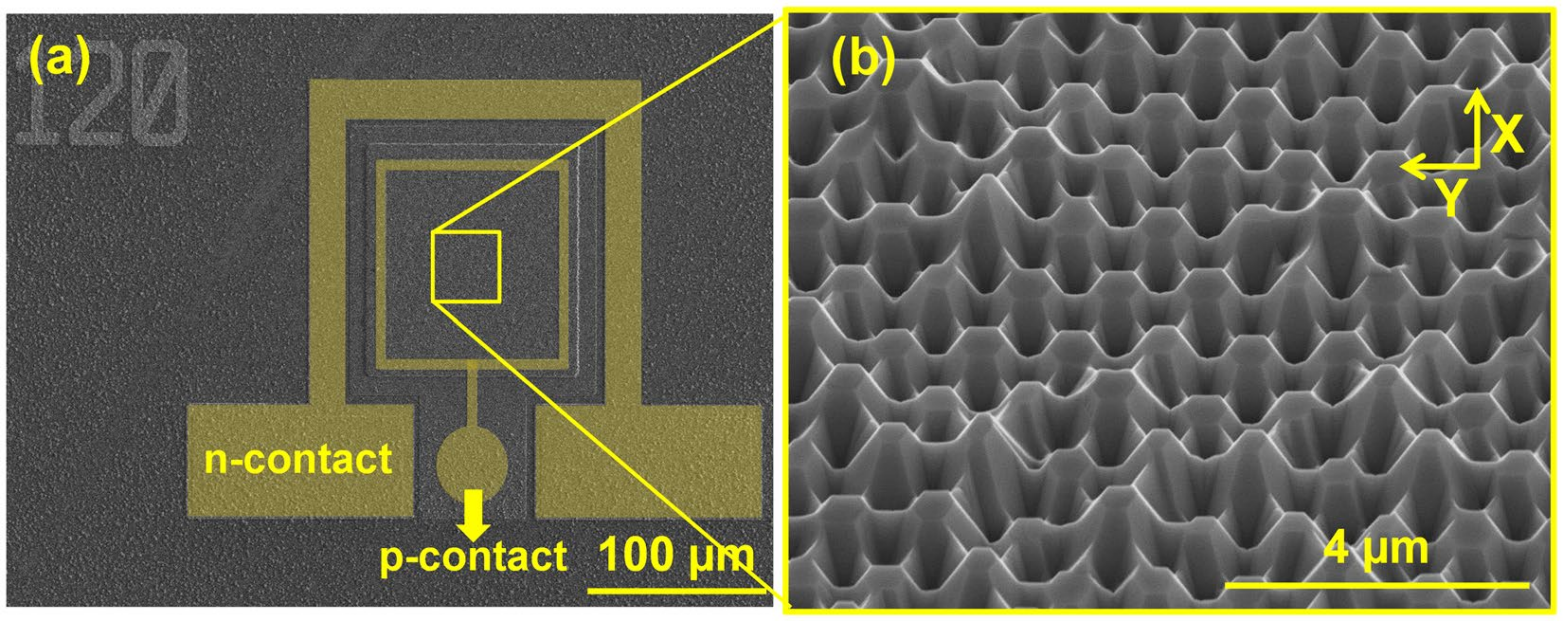
(**a**) SEM image of the nanowire-based LEDs after quantum well and p-GaN growth. (**b**) SEM image of fabricated nanowire-based LED with n-contact and p-contact.

After developing the growth conditions for the GaN nanowire cores (Fig. 3a), a 25 nm underlayer of aluminum gallium nitride (AlGaN) was grown on the GaN cores, followed by a 25 nm cap layer of GaN using the pulsed-mode technique (Fig. 3b). The AlGaN underlayer was previously shown to significantly reduce the reverse-leakage current in nanostructure LEDs by suppressing the incorporation of impurities during the p-GaN growth^78^. Four pairs of InGaN/GaN quantum wells were grown around the cap layer, followed by a 200 nm p-GaN layer grown using continuous-mode MOCVD. The AlGaN underlayer and cap layer were grown at 930 °C and a pressure of 90 Torr. The quantum well, barrier, and p-GaN were grown at 740 °C, 780 °C, and 920 °C respectively. The pressure was held at 200 Torr during the quantum well, barrier, and p-GaN growth. Figure 4a shows the nanowires after the quantum well and p-GaN growth. The supporting information document provides more details about the processing steps and epitaxial growth. Figure 4b shows an SEM image of a fabricated nanowire-based LED. To obtain uniform lateral current spreading, a 200-nm-thick layer of indium tin oxide (ITO) was deposited on the LEDs. Ti/Al/Cr/Au and Cr/Au were used as n-contacts and p-contacts, respectively.

## Optical Characterization and Study of Carrier Dynamics

**Figure 5:**
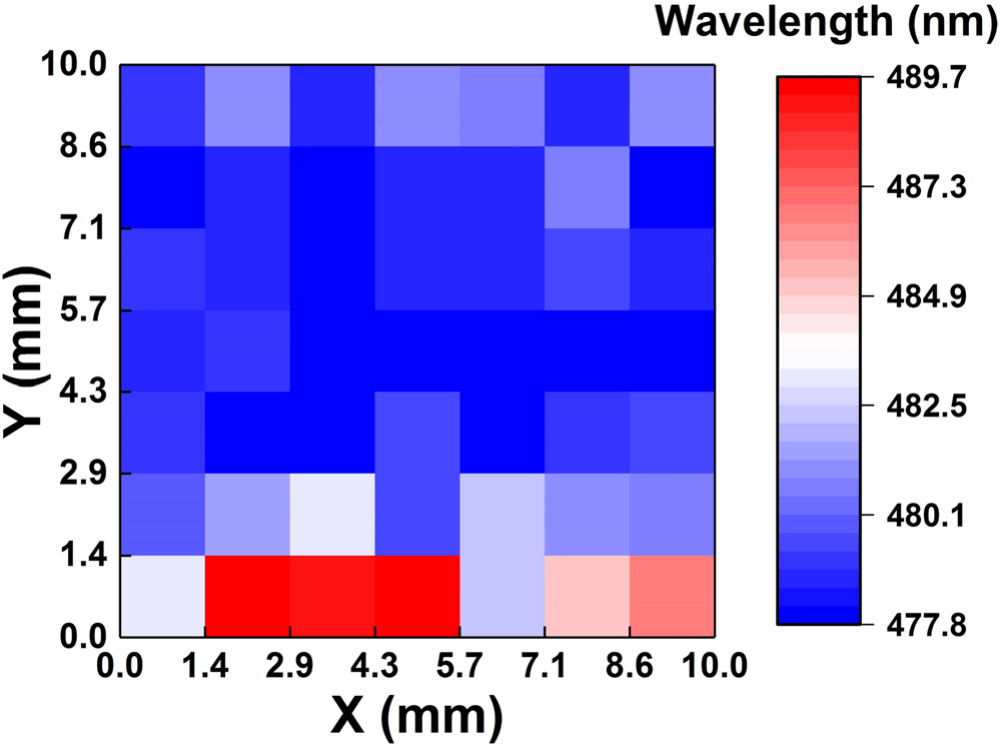
PL peak wavelength measured at 49 points (an array of 7x7 points) over a 1 cm x 1 cm area of the sample.

**Figure 6:**
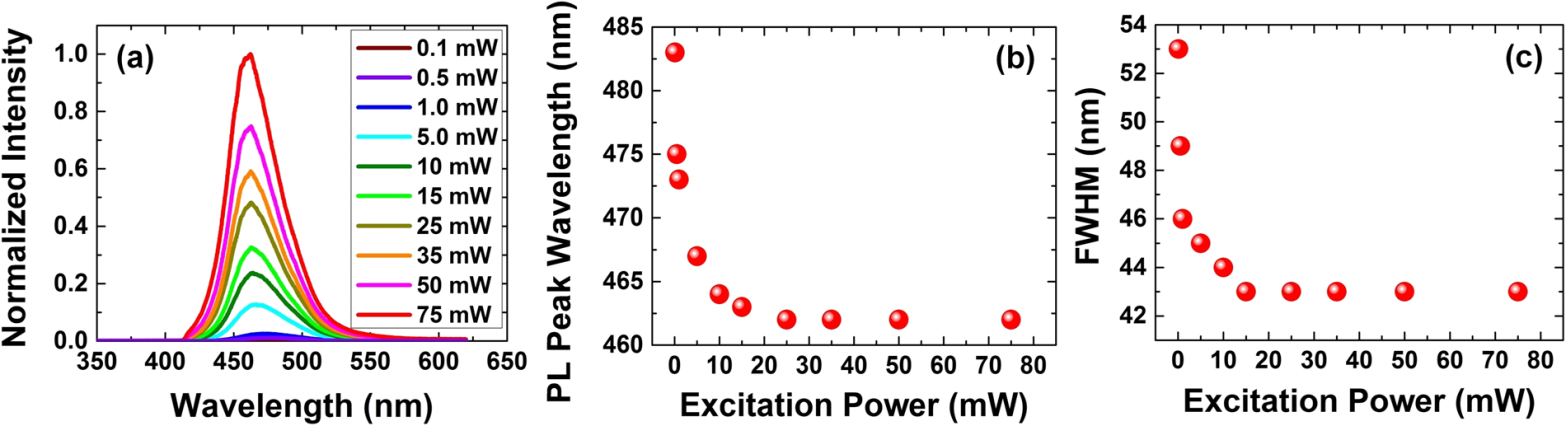
(**a**) PL spectra at different excitation powers (**b**) PL peak wavelength at different excitation powers (**c**) PL FWHM at different excitation powers.

Initially the PL characteristics of the nanowire LEDs were measured to evaluate the fundamental active region quality in the absence of electrical injection effects. Micro-photoluminescence (µPL) measurements were performed using 405 nm excitation from a frequency-doubled Ti:Sapphire laser. A long working distance (50x) micro-objective was used to excite a circular area with a diameter of ~10 µm. The pumping wavelength of 405 nm ensures photo-generation of carriers only within the active region and enables uniform pumping of the quantum wells. Additional details about the µPL are given in the accompanying supporting information. To understand the uniformity of the QW growth over the sample area, PL measurements were performed on 49 points (an array of 7 × 7 points) over a 1 × 1 cm area (most of the sample). The PL peak wavelength map is shown in Fig. 5. Some non-umiformity is observed on the bottom edge of the wafer, but most of the sample exhibits uniform PL near 480 nm. The non-uniformity observed along the bottom edge is due to the placement and orientation of the sample on the growth susceptor in our rotating disc reactor, which creates a non-uniform temperature profile on the sample. The sample is placed within a shallow cut-out in the susceptor that is larger than the sample, with the bottom edge being in contact with a sidewall of the cut-out, resulting in a lower temperature along the bottom edge of the sample. Since indium incorporation is very sensitive to temperature and higher for lower temperatures, longer wavelength emission results from LEDs near the bottom edge. The room-temperature (RT) µPL as a function of laser excitation power is shown in Fig. 6a. The µPL peak wavelength and full-width at half maximum (FWHM) are shown in Fig. 6b and c, respectively. The absence of defect-related yellow-band emission in the µPL spectra is indicative of high-quality, bright quantum wells. As the excitation power increases from 0.1 to 75 mW, the µPL peak wavelength shifts from 483 nm to 462 nm and the FWHM decreases from 53 nm to 43 nm. For nonpolar active regions in nanowires, the blueshift in the PL peak wavelength is attributed to the band-filling effect and non-uniform indium distribution along the nanowires^82,83^.

**Figure 7:**
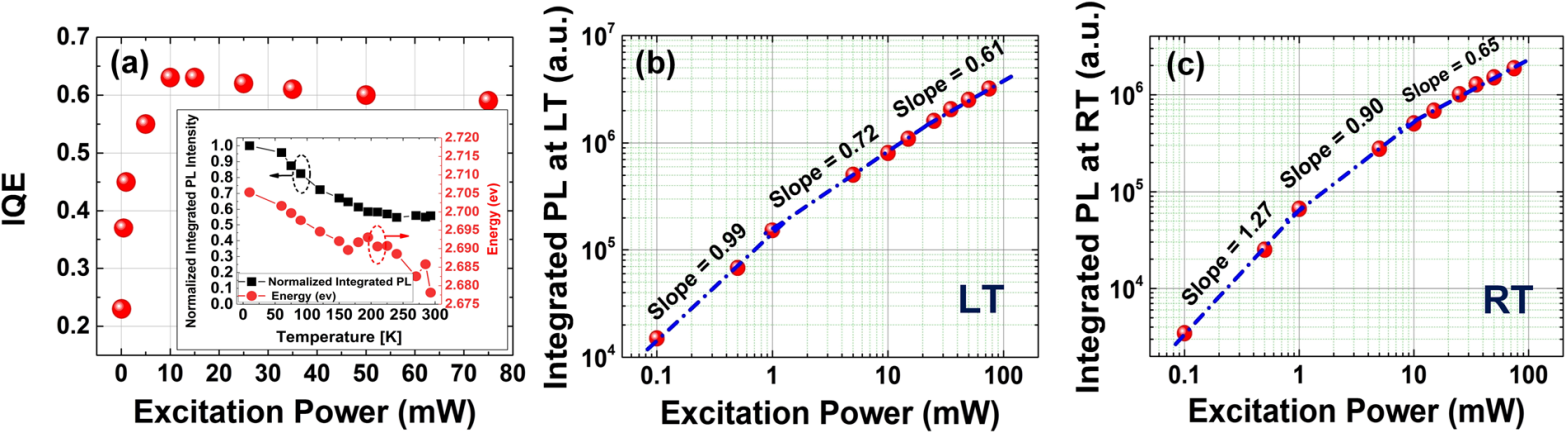
Optical characterization of GaN/InGaN core-shell LED (**a**) IQE measurement using low temperature/room temperature integrated PL technique. The inset shows the normalized integrated PL intensity and emission energy vs. temperature. (**b**) Integrated PL vs. excitation power in log-log scale at low temperature. (**c**) Integrated PL vs. excitation power in log-log scale at room temperature.

To further understand the quality of the quantum wells, the internal quantum efficiency (IQE) of the LEDs was measured. We applied the conventional method of dividing the integrated µPL at room temperature (RT) by that at low temperature (LT) −10 °K- to measure the IQE at different excitation powers^84^. Figure 7a shows the measured IQE versus excitation power, and the inset shows the temperature dependence of the normalized integrated µPL intensity and µPL peak energy measurement at an excitation power of 10 mW. The IQE versus excitation power plot provides important information about the efficiency droop for the nanowire-based LED. Previously, other groups only reported the peak IQE, which ranged from 8% to 58% in various studies^44,45,77,83^. The peak IQE here is 62%, which is among the highest reported for GaN/InGaN core-shell nanowire-based LEDs. Figures 6c and 7b show the integrated PL versus excitation power at LT and RT, respectively. At LT (10 °K) the slope of the integrated PL vs. excitation power is close to 1 for excitation powers less than 1 mW, which indicates radiative recombination is dominant^85^. The slope decreases to 0.61 at higher excitation powers. Even at LT, a sub-linear slope at higher excitation powers has been observed^86^ and can be attributed to either Auger recombination^87,88^, absorption saturation^84,89,90^, or generation of hot carriers^91^. The integrated PL versus excitation power at RT has a slope of 1.27 for excitation powers below 1 mW. This clearly indicates the presence of a combination of radiative and non-radiative recombination^85^. The slope approaches 1 for excitation powers ranging from 1–10 mW, which suggests the radiative recombination rate has become dominant over the non-radiative rate^85^. The slope decreases to 0.65 for excitation powers greater than 10 mW, indicating Auger recombination or carrier leakage has become dominant^85,92^. The change in the slope of the integrated PL (see Fig. 6c) at 10 mW directly corresponds with the peak of the IQE. The IQE is maximum at 10 mW and exhibits efficiency droop at higher excitation powers.

**Figure 8:**
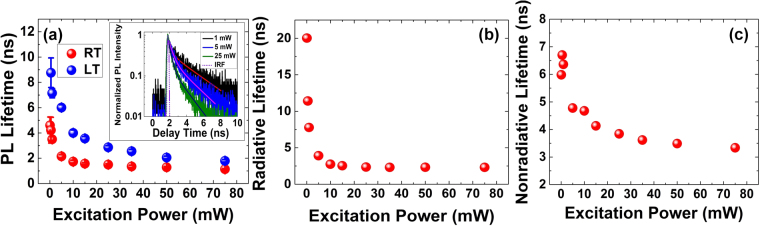
Optical characterization of GaN/InGaN core-shell LED (**a**) PL lifetime measured at room temperature and low temperature. (**b**) Radiative lifetime at room temperature. (**c**) Nonradiative lifetime at room temperature.

In addition to high quantum efficiency, the response time of the LEDs is critical for their implementation in displays, VLC, and Li-Fi technologies. The carrier recombination rate is a key parameter in determining the response time. Time-resolved photoluminescence (TRPL) was used to measure the carrier lifetime of the nanowire-based LEDs at RT and LT for excitation powers ranging from 0.1 mW to 75 mW. More details about this measurement are given in the supporting information. The PL transients were fit using the bi-exponential decay function, ${A}_{1}{e}^{-t/{\tau }_{1}}+{A}_{2}{e}^{-t/{\tau }_{2}}$, where A_1_ and A_2_ are amplitudes and τ_1_ and τ_2_ are the time constants of the fast decay and slow decay components, respectively. The slow decay component is considered the PL lifetime^93–96^. Fig. 8a shows the PL lifetimes (extracted from the slow decay component) versus excitation power at RT and LT. The inset of Fig. 8a shows a few examples of the RT PL transients for excitation powers of 1, 5, and 25 mW. The instrument response time (IRF) is also shown and verifies it is much shorter than the measured lifetimes. The PL lifetime measured for the nanowire-based LED shows a minimum of 1.3 ns, This lifetime is at least 3 times shorter than that of typical planar c-plane LEDs, which are in the range of 4–20 ns at high excitation powers^51,52^. The shorter lifetime in the nanowire LEDs is mostly attributed to the higher electron-hole wave function overlap for QWs grown on the m-plane side walls of the nanowires, rather than non-radiative surface recombination since the p-GaN is ~200 nm thick. Shorter carrier lifetimes provide the possibility of higher 3dB bandwidth.

Figure 8a shows the PL lifetime decreases as the excitation power increases both at RT and LT. Also, the PL lifetime at LT is longer than that at RT for all excitation powers. The difference between the PL lifetime at LT and the PL lifetime at RT is highest at low excitation powers. At low excitation powers and LT, radiative recombination is dominant (slope ≈ 1 in Fig. 6b), while at RT for the same excitation powers both radiative and non-radiative recombination exist (slope > 1 in Fig. 6c). The combination of radiative and non-radiative recombination lowers the PL lifetime at RT.

Having obtained the IQE and PL lifetime data at RT, the radiative and non-radiative lifetimes can be decoupled using equations (1) and (2),

1 $${\tau }_{R}={\tau }_{PL}/IQE$$

2 $${\tau }_{NR}={\tau }_{PL}/(1-IQE)$$

where ${\tau }_{{PL}}$ is the PL lifetime, ${\tau }_{{R}}$ is the radiative lifetime, and ${\tau }_{{NR}}$ is the non-radiative lifetime. Fig. 8b and c show the radiative and non-radiative lifetimes versus excitation power, respectively. As the excitation power increases from 0.1–10 mW, the radiative lifetime decreases. For excitation powers beyond 10 mW, the radiative lifetime remains fairly constant. The onset of the constant radiative lifetime above 10 mW in Fig. 8b corresponds to the point at which the slope of the integrated PL versus excitation power changes to 0.65 in Fig. 7c. The non-radiative lifetime in Fig. 8c continues to decrease for excitation powers above 10 mW. At high excitation powers, both Auger recombination and carrier leakage are present based on the slope $=0.65$ in Fig. 7c. Auger recombination is the dominant process in reducing the non-radiative lifetime in Fig. 8c. Carrier leakage opposes this reduction in non-radiative lifetime and eventually leads to the saturation of the non-radiative lifetime at higher excitation powers.

## Electrical Characterization

**Figure 9:**
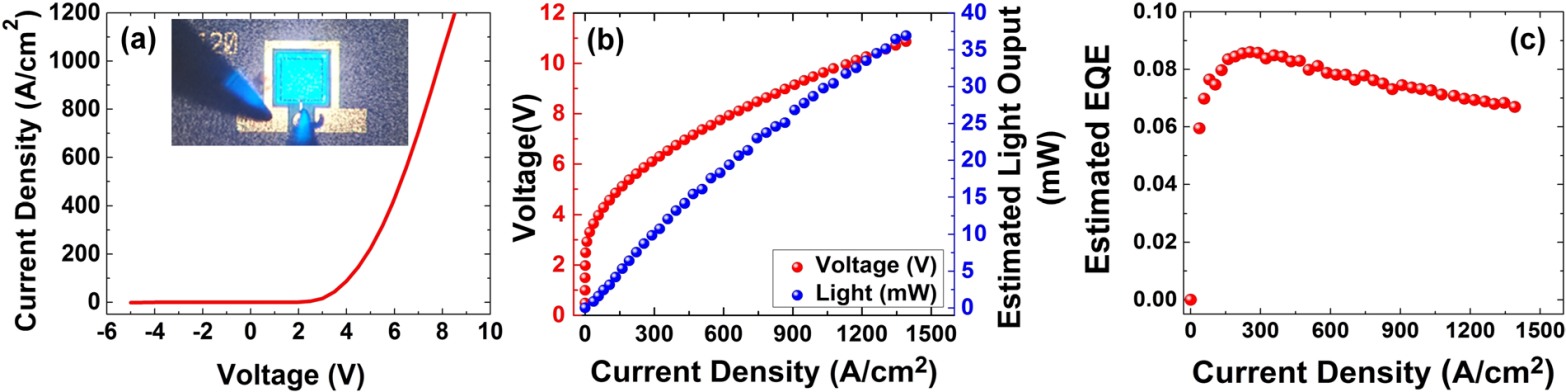
(**a**) J-V plot. Inset image shows an operating LED with 120 μm by 120 μm mesa size (**b**) L-J-V curve for a device with 120 μm by 120 μm mesa size (**c**) Estimated EQE.

**Figure 10:**
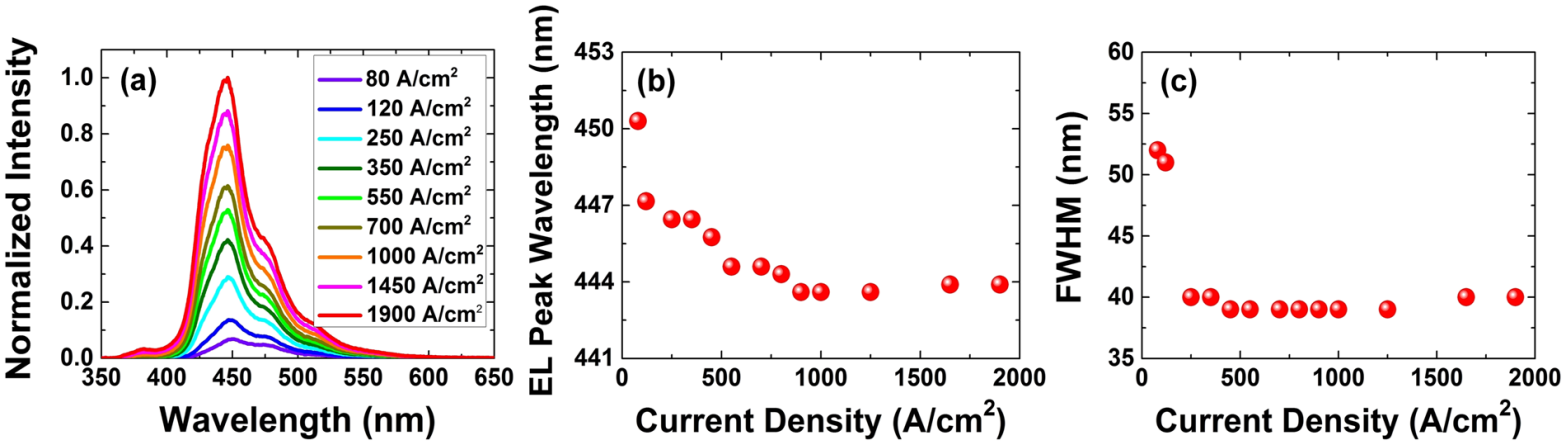
(**a**) EL spectra at different current densities, (**b**) EL peak wavelength at different current densities, (**c**) FWHM at different current densities.

While optical characterization techniques provide useful information for understanding the quantum well quality, these techniques do not provide information on the properties of the LED under electrical injection. These properties play a critical role in the performance of nanowire-based LEDs and include carrier transport, reverse-leakage current, series resistance, and turn-on voltage. Figure 9a shows the continuous-wave J-V plot for an LED with a 120-µm mesa size. The current density (J) is calculated based on the planar footprint. If the effective nanowire surface area is used to calculate the current density, the current density would be reduced by a factor of 2.3 in Figs 9 and 10. Figure 9b and c show the light–current density–voltage (L–J–V) characteristics and estimated EQE under room-temperature pulsed operation (2% duty cycle, 2 µs pulse width), respectively. The J-V plot does not exhibit any reverse leakage current. The LED shows a turn-on voltage of 2.9 V and a series resistance of 25 Ω. The combination of a low turn-on voltage and low series resistance places this device among the highest performing of those reported for GaN/InGaN core-shell nanowire-based LEDs in terms of current-voltage characteristics and internal quantum efficiency^36,44,45,77^. With advanced LED packaging techniques unavailable for this work, the light extraction efficiency (EXE) was simulated to enable an estimate of the total output power and external quantum efficiency (EQE) of the device. A commercial-grade simulator based on the finite-difference time-domain (FDTD) method (Lumerical FDTD Solutions) was used to calculate the EXE of the nanowire-based LED. The FDTD method is a fully vectorial approach that naturally gives both the time domain and frequency domain information^97^. An EXE of 13.2% from the top surface was calculated for the nanowire-based LED, which is higher than the simulated EXE (8.1%) from the top surface of a planar LED. The simulation methods are explained in more detail in the supporting information. Assuming an injection efficiency (IE) of 1, the total extracted power from the top surface of the LED (P) was calculated using equation (3). Here, *h* is Plank’s constant, υ is the LED electroluminescence (EL) emission frequency, *q* is electron charge, and *I* is current. In this calculation, we assume that the peak IQE coincides with the peak of the EQE (i.e., EXE does not depend upon injection current)

3 $$P=EQE\frac{h\upsilon }{q}I$$

where,

4 $$EQE=EXE\times IQE$$

Figure 10a,b and c show the electroluminescence (EL) spectra, peak wavelength, and FWHM at different current densities, respectively. As the current density increases from 80 A/cm^2^ to 1.9 kA/cm^2^, the EL peak wavelength exhibits a blueshift from 452 nm to 444 nm. The shift is attributed to non-uniformities in the indium incorporation in different regions of the nanowires^36,45,82,83^. Large blueshifts in the EL peak wavelength of nanowire-based LEDs, ranging from 62 nm to 180 nm, have been observed by other research groups^36,98^. As the current density increases from 80 to 500 A/cm^2^ the FWHM of the EL spectra decreases from 52 nm to 38 nm. The FWHM remains fairly constant from 500 A/cm^2^ to 1.25 kA/cm^2^ and increases to 40 nm for higher current densities. Although we were unable to perform burn-in measurements on these samples due to lack of packaging capabilities, this will be the subject of future work.

**Figure 11:**
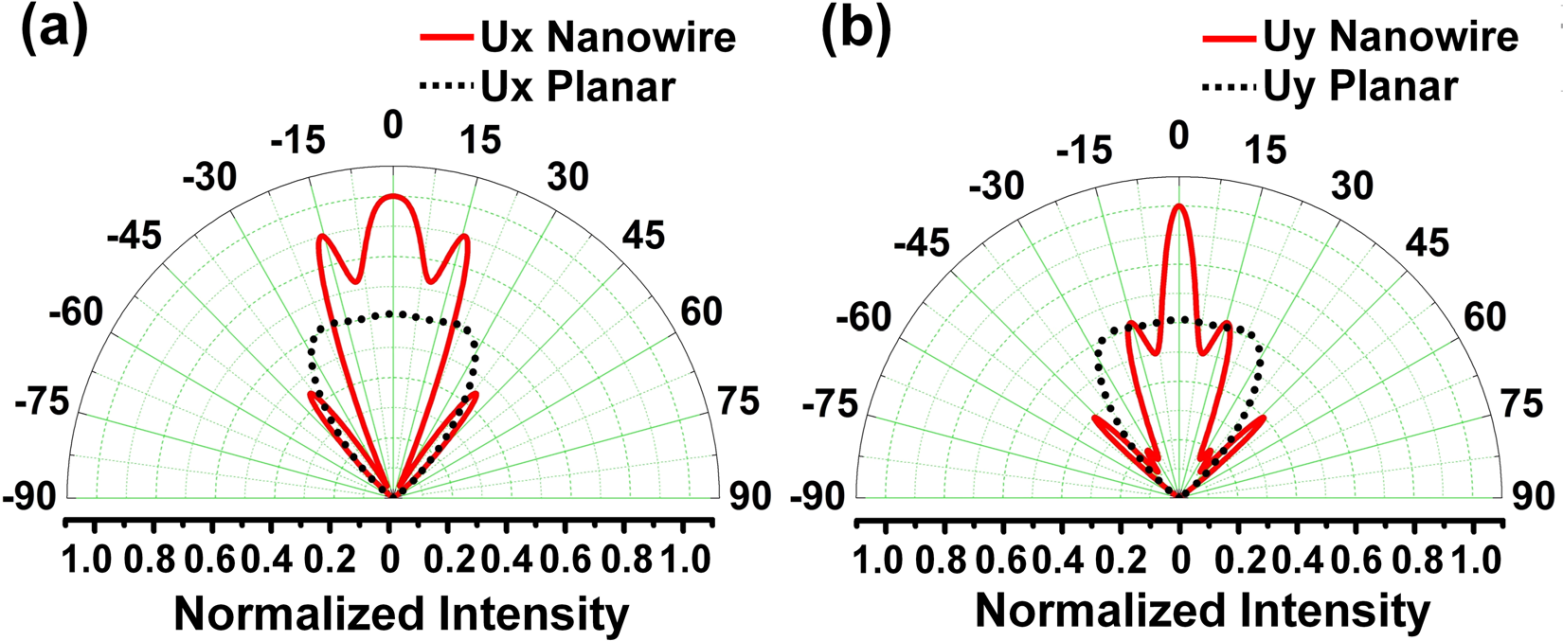
Simulated angular distribution of extracted light for nanowire-based LED along (**a**) x-axis (**b**) Yaxis.

The far-field emission pattern of µ-LEDs has an important role in their performance. A relatively directional output power, ranging from ±15–20°, is preferred for full-color displays^6,7^. Such far-field emission patterns have been achieved by utilizing the photonic crystal effect for conventional planar structures^10–12^. FDTD-Lumerical was used to simulate the far-field emission pattern of the nanowire-based LED structure shown in Fig. 4a. The angular distributions of extracted light along the x and y-axes are shown in Fig. 11a and b. These angular distributions exhibit multiple lobes and a relatively directional emission pattern compared to the Lambertian-type far-field pattern of conventional planar LEDs^99,100^. The higher directionality is attributed to the periodic nanowire structure, which enhances the diffracted power normal to the LED surface. A directional far-field emission pattern ranging between ±15° is predicted for our nanowire-based LED. Directional far-field emission patterns in the range of ${\rm{\pm }}$30° and below have been achieved by other research groups using nanowire-based LEDs^7,13^. This unique property makes nanowire-based LEDs a good candidate for the next generation of high brightness displays. We note that no extra PhC patterning or micro lenses are needed to obtain this relatively directional emission pattern from nanowire-based LEDs.

## Conclusion

In this work, we demonstrated high-efficiency electrically injected GaN/InGaN core-shell nanowire-based LEDs using bottom-up selective-area epitaxy. The electrical and optical properties of the LEDs were studied in detail. The LEDs showed high IQE (62%), low turn-on voltage (2.9 V), low series resistance (25 Ω), and short carrier lifetimes (1–2 ns). These results are among the highest performance levels for nanowire-based LEDs thus far. In addition, FDTD modeling revealed that the nanowire-based LEDs have a strongly directional far-field emission pattern. Properties such as high IQE, short carrier lifetime, and emission directionality are attractive for solid-state lighting, visible-light communication, and μ-LED displays, respectively. While the performance level of nanowire-based LEDs is still below that of planar LEDs, nanowire-based LEDs offer unique properties (e.g., monolithic RGB emission and directionality) that are expected to be beneficial for some specific applications (e.g., μ-LED displays).

## Electronic supplementary material

Supporting information
