# Supplementary material for: Carrier Dynamics and Electro-Optical Characterization of High-Performance GaN/InGaN Core-Shell Nanowire Light-Emitting Diodes
Source: Sci Rep. 2018 Jan 11;8:501. doi: 10.1038/s41598-017-18833-6 (PMC5764991; doi:10.1038/s41598-017-18833-6)
Supplement: Supplementary file 1 — Supporting information [file 41598_2017_18833_MOESM1_ESM.pdf]

Supporting Information

Carrier Dynamics and Electro-Optical Characterization of High-Performance GaN/InGaN Core-Shell Nanowire Light-Emitting Diodes

Mohsen Nami,*† Isaac E. Stricklin,† Kenneth M. DaVico,† Saadat Mishkat-Ul-Masabih, † Ashwin K. Rishinaramangalam,† S. R. J. Brueck,† Igal Brener,‡† and Daniel F. Feezell†

†Center for High Technology Materials, University of New Mexico, Albuquerque, USA

‡Center for Integrated Nanotechnologies, Sandia National Laboratories, Albuquerque, USA

KEYWORDS: Nanowire, Core-shell, GaN/InGaN, Selective-area epitaxy, MOCVD, Light-emitting-diodes.

1: Processing Steps for GaN/InGaN Core-Shell Nanowire LEDs

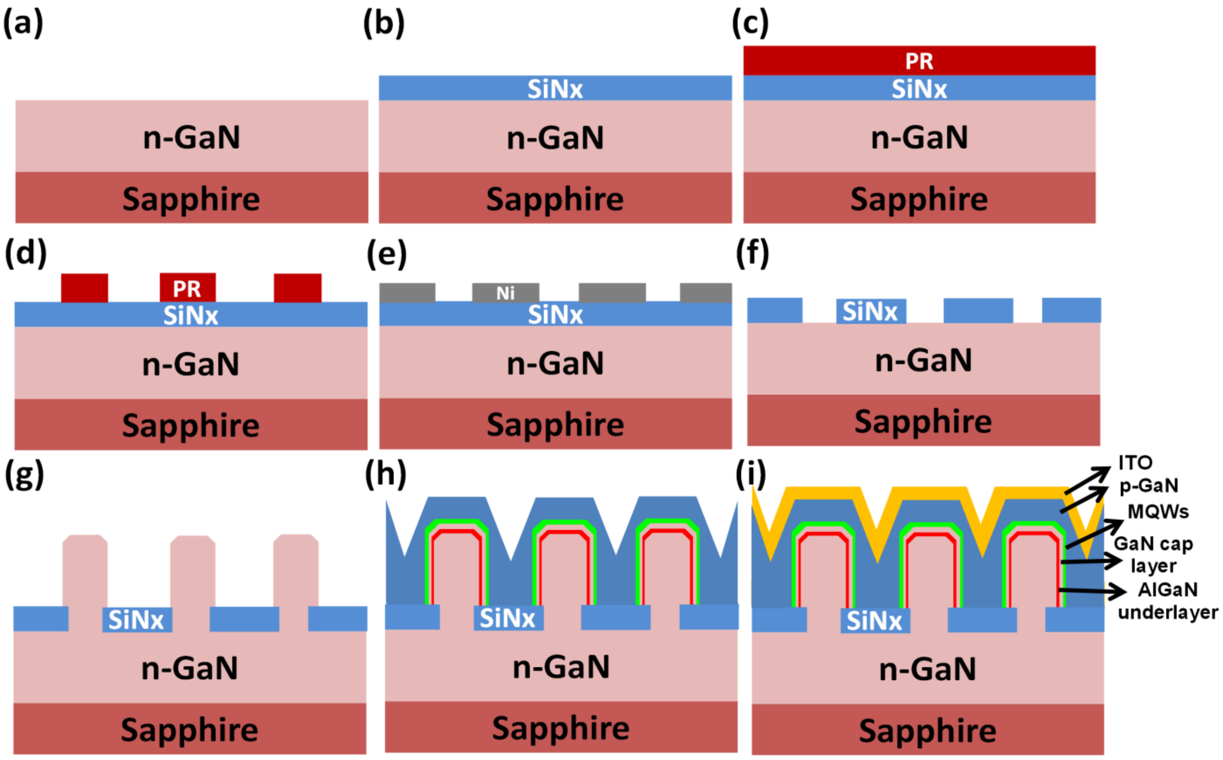

Figure 1: Processing steps for GaN/InGaN core-shell LEDs. (a) 2 μm GaN template on Sapphire. (b) Deposition of 120 nm of SiN_x using plasma-enhanced chemical vapor deposition of SiN_x. (c) Spin photoresist. (d) Interferometric lithography. (e) Ni deposition and lift-off. (f) Reactive ion etching and piranha cleaning. (g) Growth of GaN nanowires using pulsed MOCVD. (h) Growth of AlGaN underlayer, quantum wells, and p-GaN using conventional continuous MOCVD. (i) ITO deposition.

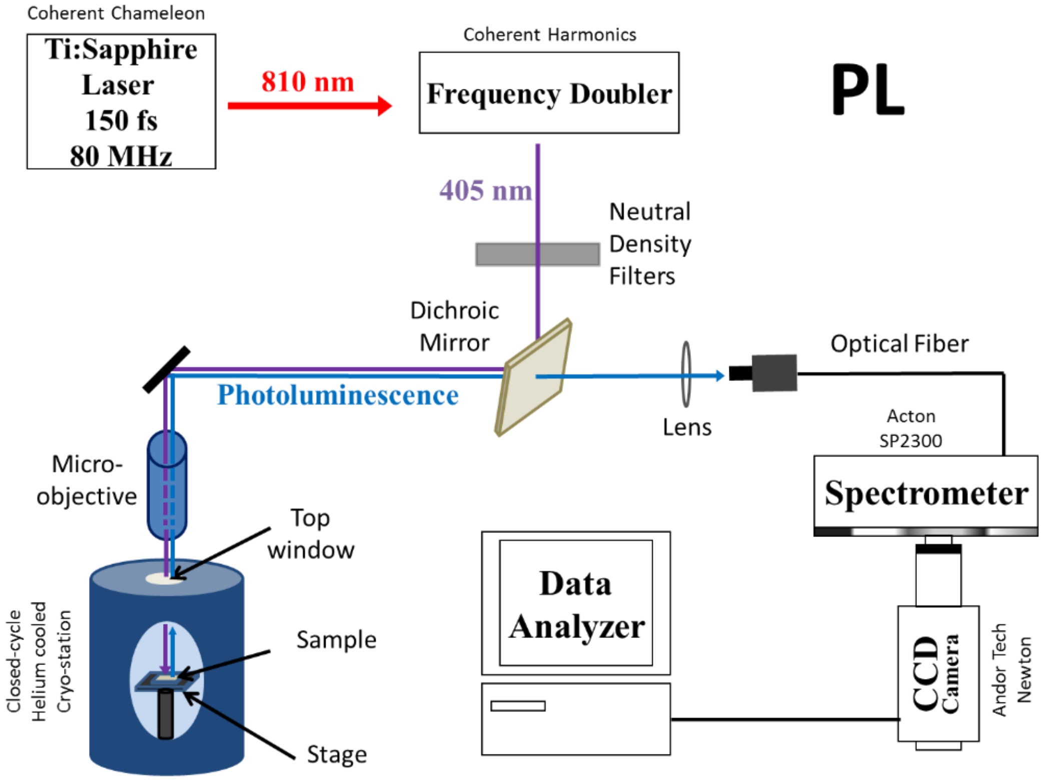

Figure 2: Photoluminescence set-up.

Figure 2 shows the experimental setup for conducting the micro-photoluminescence (μ-PL) measurements of the nanowire-based LEDs. A Coherent Chameleon titanium-sapphire pulsed laser, tuned to 810 nm with pulse widths of 140 fs and a repetition rate of 80 MHz, was used to excite the LEDs. The tunable range of the laser is 680 nm to 1080 nm, so frequency doubling by a Coherent Harmonics Generator frequency doubler was required to obtain the excitation wavelength of 405 nm. From the frequency doubler, the laser light was passed through a continuous neutral density filter to select excitation powers of 0.1 mW, 0.5 mW, 1 mW, 5 mW, 10 mW, 15 mW, 25 mW, 35 mW, 50 mW and 75 mW. A Semrock high-pass 405 nm dichroic mirror directed this reduced beam towards a Montana Instruments cryostation which housed the nanowire-based LED. The cyrostation was used to control the temperature of the nanowire-based LED from 293 K to 10 K during the measurements. A long working distance micro-objective (50x) focused the beam onto the nanowire-based LED in a circular spot approximately 10 μm in diameter. The photoluminescence emitted from the nanowire-based LED was composed of wavelengths longer than 405

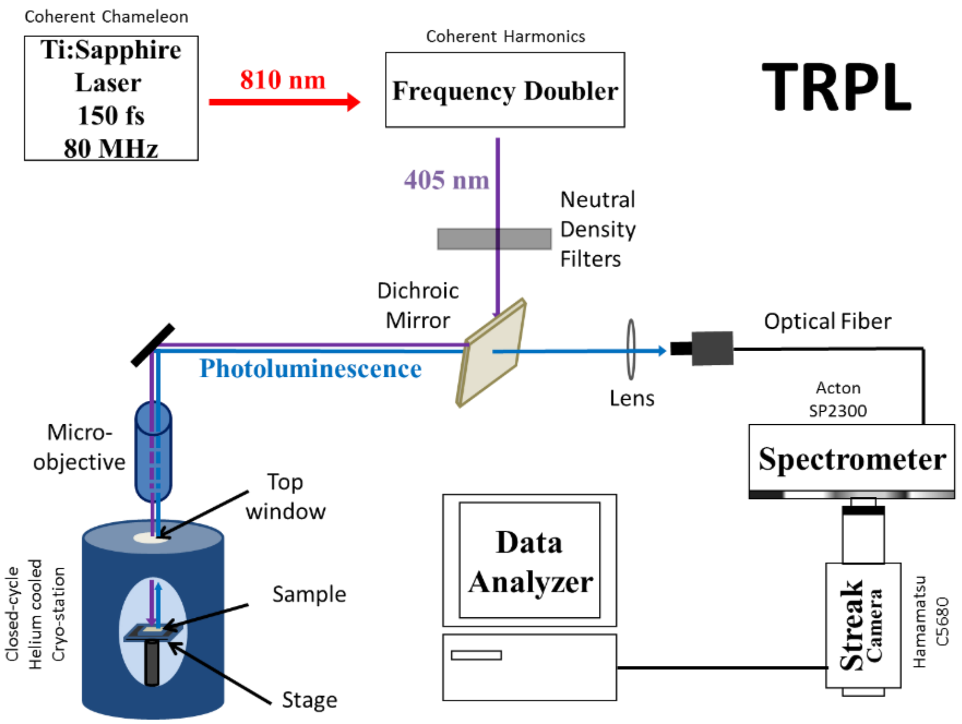

Figure 3: Time-Resolved Photoluminescence set-up.

nm which could pass through the dichroic mirror. The photoluminescence was focused through a lens into an optical fiber, and coupled into an Acton SP2300 Spectrometer. An Andor Tech Newton CCD camera then recorded the intensity spectrum from the spectrometer.

The time-resolved photoluminescence (TRPL) measurement setup is shown in Figure 3. The excitation source was a Coherent Chameleon Ti:Sapphire pulsed laser with a repetition rate of 80 MHz, tuned to 810 nm, and its output frequency doubled to 405 nm. The 405 nm emission was reflected by a dichroic mirror to a micro-objective lens, and was focused onto the nanowire-based LED. After the excitation of the nanowire-based LED, the photoluminescence passed through the dichroic mirror, a lens, the Acton SP2300 Spectrometer, and into a Hamamatsu C5680 streak camera. The streak camera was equipped with the Hamamatsu M5677 Blank Unit to measure the time variation of the intensity of the photoluminescence over a time range of 10 nanoseconds. To obtain the instrument response function (IRF) of the streak camera to the excitation source, a TRPL measurement was performed on scattered light originating from the 405 nm laser beam.

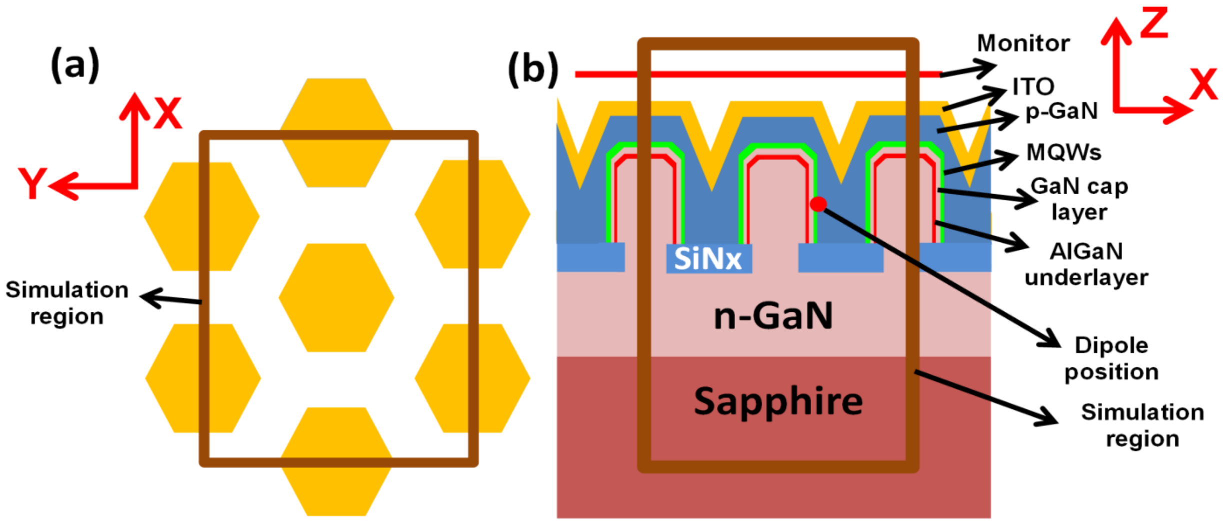

Figure 4: Simulation of GaN/InGaN core-shell nanowire-based LED. (a) Schematic of the top view of the simulated nanowires and the simulation region. (b) Schematic of the cross section of the nanowire-based LEDs, different layers, top monitor to calculate extraction efficiency, and simulation region.

3: Simulation Method:

A commercial-grade simulator based on the finite-difference time-domain (FDTD) method (Lumerical FDTD Solutions) was used to calculate the EXE of the nanowire-based LED. The geometry of the nanowire-based LEDs was extracted from the SEM images of the GaN nanowires before and after the AlGaN underlayer growth, as well as after the p-GaN growth. A cross section of the nanowires, and the simulation region used, are shown in Figures 4.a and 4.b. A simulation region of 2 µm by 1.7 µm was selected as a unit cell. Perfectly matched layer (PML) boundary conditions were used for the top z-axis boundary condition, periodic boundary conditions were used for x and y axes, and metal boundary conditions were used for the bottom z-axis. To represent the quantum well emission, we used the common approach of incorporating a radiating electric dipole source at the location of the active region.[1] The emission from nonpolar InGaN quantum wells is well modeled by a single dipole source directed along the a-direction of the wurtzite crystal (Y-direction in Fig. 4(b)).[2,3] Six simulations were performed for each dipole at each side of the nanowire-based LED. The results were added incoherently to find the extraction efficiency and far-field emission pattern of the LED using a monitor at the top of the structure.

References:

1. Born, M. Atomic Physics, 1969, Blackie & Son, 8th ed.

2. Matioli, E.; Brinkley, S:, Kelchner, K.M.; Nakamura, S.: DenBaars, S.; Speck, J.; Weisbuch, C.. Polarized light extraction in m-plane GaN light-emitting diodes by embedded photonic-crystals. *Applied Physics Letters*, **2011**, *98*(25), 251112.

3. Gardner, N.F.; Kim, J.C.; Wierer, J.J.; Shen, Y.C.; Krames, M.R. Polarization anisotropy in the electroluminescence of m-plane InGaN–GaN multiple-quantum-well light-emitting diodes. *Applied Physics Letters*, **2005**, *86*(11), 111101.
